# Readmission and mortality in patients ≥70 years with acute myocardial infarction or heart failure in the Netherlands: a retrospective cohort study of incidences and changes in risk factors over time

**DOI:** 10.1007/s12471-019-1227-4

**Published:** 2019-02-04

**Authors:** P. Jepma, G. ter Riet, M. van Rijn, C. H. M. Latour, R. J. G. Peters, W. J. M. Scholte op Reimer, B. M. Buurman

**Affiliations:** 1 ACHIEVE Centre for Applied Research, Faculty of Health, Amsterdam University of Applied Sciences, Amsterdam, The Netherlands; 2 Amsterdam UMC, Department of General Practice, University of Amsterdam, Amsterdam, The Netherlands; 3 Amsterdam UMC, Department of Internal Medicine, Section of Geriatric Medicine, University of Amsterdam, Amsterdam, The Netherlands; 4 Amsterdam UMC, Department of Cardiology, University of Amsterdam, Amsterdam, The Netherlands

**Keywords:** Acute myocardial infarction, Heart failure, Mortality, Patient readmission, Transitional care

## Abstract

**Objectives:**

To determine the risk of first unplanned all-cause readmission and mortality of patients ≥70 years with acute myocardial infarction (AMI) or heart failure (HF) and to explore which effects of baseline risk factors vary over time.

**Methods:**

A retrospective cohort study was performed on hospital and mortality data (2008) from Statistics Netherlands including 5,175 (AMI) and 9,837 (HF) patients. We calculated cumulative weekly incidences for first unplanned all-cause readmission and mortality during 6 months post-discharge and explored patient characteristics associated with these events.

**Results:**

At 6 months, 20.4% and 9.9% (AMI) and 24.6% and 22.4% (HF) of patients had been readmitted or had died, respectively. The highest incidences were found in week 1. An increased risk for 14-day mortality after AMI was observed in patients who lived alone (hazard ratio (HR) 1.57, 95% confidence interval (CI) 1.01–2.44) and within 30 and 42 days in patients with a Charlson Comorbidity Index ≥3. In HF patients, increased risks for readmissions within 7, 30 and 42 days were found for a Charlson Comorbidity Index ≥3 and within 42 days for patients with an admission in the previous 6 months (HR 1.42, 95% CI 1.12–1.80). Non-native Dutch HF patients had an increased risk of 14-day mortality (HR 1.74, 95% CI 1.09–2.78).

**Conclusion:**

The risk of unplanned readmission and mortality in older AMI and HF patients was highest in the 1st week post-discharge, and the effect of some risk factors changed over time. Transitional care interventions need to be provided as soon as possible to prevent early readmission and mortality.

**Electronic supplementary material:**

The online version of this article (10.1007/s12471-019-1227-4) contains supplementary material, which is available to authorized users.

## What’s new?

- The highest incidences of readmission and mortality in older patients with acute myocardial infarction (AMI) or heart failure (HF) were found in week 1. The timing of transitional care interventions needs to be improved to prevent early events.
- Patients with comorbidities, an admission in the previous 6 months, patients living alone and non-native Dutch patients were at highest risk for early readmission and mortality.
- Better alignment of transitional interventions is needed, as risk factors and timing of readmission and mortality differ for older AMI and HF patients. Therefore, disease-oriented treatments in transitional interventions might be integrated to reduce risks.

## Introduction

Older patients who have been recently discharged after hospital admission for cardiac events are at high risk of readmission and mortality [1]. Research has shown that factors such as higher age [2, 3], the presence of comorbidities [4, 5], being single [2], low socioeconomic status [2, 6] and an admission in the previous 6 months [7] increase the risk of readmission and mortality.

Transitional care interventions (TCIs) aim to improve continuity of care after discharge through multidisciplinary collaboration, structured discharge planning and early follow-up home visits and have proven to lower the risk of readmission and mortality [8–10]. The start and duration of TCIs vary. Le Berre et al. [10] found that TCIs started after an average of 7.9 days (SD 6.2) post-discharge and lasted for an average of 179.7 days (SD 158.5), showing large diversity in duration of interventions. It is currently unclear what the optimal time window is for TCIs in (various subgroups of) older cardiac patients. Better delineation of which older cardiac patients would benefit most in which time windows would allow the most efficient deployment of TCIs.

Therefore, we determined the risk of a first unplanned all-cause readmission and mortality of patients ≥70 years with acute myocardial infarction (AMI) or heart failure (HF) and explored the extent to which effects of particular baseline risk factors vary over time.

## Methods

### Data sources

We used the National Medical Registration (LMR) of 2008 (and 2009 for the follow-up) from Statistics Netherlands [11] in which 88% of all hospital admissions in the Netherlands were registered anonymously. The LMR was linked to the Dutch Population Registry (GBA), which contains demographic characteristics. Record linkage was successful in 88.9% of hospital-admitted patients [11]. The dates of death were obtained from the Causes of Mortality registry. The Integrated Income Data of Household registry (IIDH) was used to retrieve additional information about residence, living circumstances and annual income.

### Study population

Patients with an unplanned hospital admission in 2008 were included. Eligible patients were identified with help of the International Classification of Diseases, 9th revision, Clinical Modification (ICD-9-CM). Patients were eligible if they were ≥70 years old, had a discharge diagnosis of AMI (ICD-9 410) or HF (ICD-9 428) and had a length of stay ≥1 day. The first cardiac admission that met these criteria was considered as the index admission. Transfers to other hospitals or wards during this admission were taken as part of the same admission. No approval of the Medical Ethics Committee was necessary as data were used from national registries with anonymous information.

### Outcomes and risk factors

We examined the cumulative weekly incidence of a first unplanned all-cause readmission and mortality within 6 months. We identified potential risk factors at baseline and examined the extent to which their associations with the outcomes varied over time. An unplanned all-cause readmission was defined as any non-elective admission occurring at least 1 day after discharge from the index admission in any hospital. Risk factors were selected based on availability in the LMR, GBA and IIDH registries (Electronic Supplementary Material: Table S1–S4).

### Statistical analysis

We described data using counts and percentages for categorical variables and means with standard deviations (SD) or medians with interquartile ranges (IQR) as appropriate.

We calculated the cumulative weekly incidence for unplanned all-cause readmission and mortality in AMI and HF patients until 6 months post-discharge. The number of events per week post-discharge was divided by the number of persons at risk at the start of that week. Follow-up ended if patients experienced the event of interest, died (in case of readmission) or at 6 months after the index admission if a target event did not occur.

Then, we examined by extended multivariable Cox regression analyses [12] to what extent the effects of baseline risk factors on unplanned all-cause readmission and mortality until 6 months post-discharge varied across five time points: 3, 7, 14, 30 and 42 days post-discharge. This modified Cox regression analysis is a time-to-event analysis to study if the association of a particular factor with the outcome varies over time. It involves risk factor-time interaction terms into the regression models (dummy variables for time were coded 0 = early and 1 = late) [12]. To reduce the number of statistical tests, we performed chunk tests comparing models with and without all risk factor-time interaction terms based on the likelihood ratio test. Statistically non-significant chunk tests (*p* ≥ 0.05) were interpreted as an indication that the extended model including the interaction terms did not lead to a better fit and standard multivariable Cox regression analysis was preferred. We took statistically significant chunk tests as an indication that the model with interactions fitted the data better. We performed a stepwise backward procedure with a *p*-value for entry and removal of 0.05 and 0.10, respectively. Statistically significant risk factor-time interactions were interpreted as risk factors whose effect varied over time. We expressed all hazard ratios (HRs) such that values greater than 1 indicate higher risk at the earlier time point. HRs were displayed on a logarithmic scale to enhance compact visualisation of scattered estimates (Figs. 2, 3 and 4). Analyses were performed with SPSS Statistics 22.0 (SPSS Inc., Chicago, IL, USA).

## Results

A total of 15,012 patients ≥70 years had an unplanned hospital admission and discharge diagnosis of AMI (*n* = 5,175; 35.5%) or HF (*n* = 9,837; 65.5%). During the index admission, 1,878 patients (12.5%) died: 576 AMI patients (11.1%) and 1,302 HF patients (13.2%). Thus, a total of 13,134 patients discharged with a diagnosis of AMI (*n* = 4,599) or HF (*n* = 8,535) were included. Tab. 1 shows the patient characteristics.

**Table 1:** Baseline characteristics of included patients

	Acute myocardial infarction	Heart failure
	(*n* = 4,599)	(*n* = 8,535)
Male, *n* (%)	2,464 (53.6%)	3,749 (43.9%)
Age, mean (SD)	79.2 (6.0)	81.8 (6.3)
Native Dutch, *n* (%)	4,123 (89.6%)	7,620 (89.3%)
Patients living alone^b^, *n *(%)	1,999 (43.5%)	4,607 (54.0%)
Living in an institution, *n* (%)	314 (6.8%)	1,189 (13.9%)
Length of stay (days), median (IQR)	6.0 (4.0–10.0)	7.0 (5.0–12.0)
Admission in the previous 6 months, *n* (%)	158 (3.4%)	846 (9.9%)
*CCI*^a^ [28], *n* (%)		
– Score 1	2,933 (63.8%)	5,379 (63.0%)
– Score 2	1,200 (26.1%)	1,897 (22.2%)
– Score ≥ 3	466 (10.1%)	1,259 (14.8%)
*Annual income*^c^, *n* (%)		
– ≤€16,801	2,538 (55.2%)	4,026 (47.2%)
– >€16,801	2,059 (44.8%)	4,509 (52.8%)
*Type of hospital*, *n* (%)		
– General hospital	1,874 (40.7%)	4,482 (52.5%)
– Tertiary referral hospital	2,469 (53.7%)	4,776 (44.2%)
– University hospital	256 (5.6%)	277 (3.2%)

*IQR* interquartile range,* N* number, *SD* standard deviation

^a^Charlson Comorbidity Index (CCI) [28]: a weighted index to classify comorbid conditions based on their 1‑year mortality prognosis. The index was categorised as above. A CCI of 1 was the reference category, because acute myocardial infarction and heart failure both score 1 point in the original CCI

^b^Patients living alone or with children ≤18 years old

^c^Dichotomised, based on median income in the dataset

### Cumulative incidence of a first unplanned all-cause readmission

**Fig. 1:**
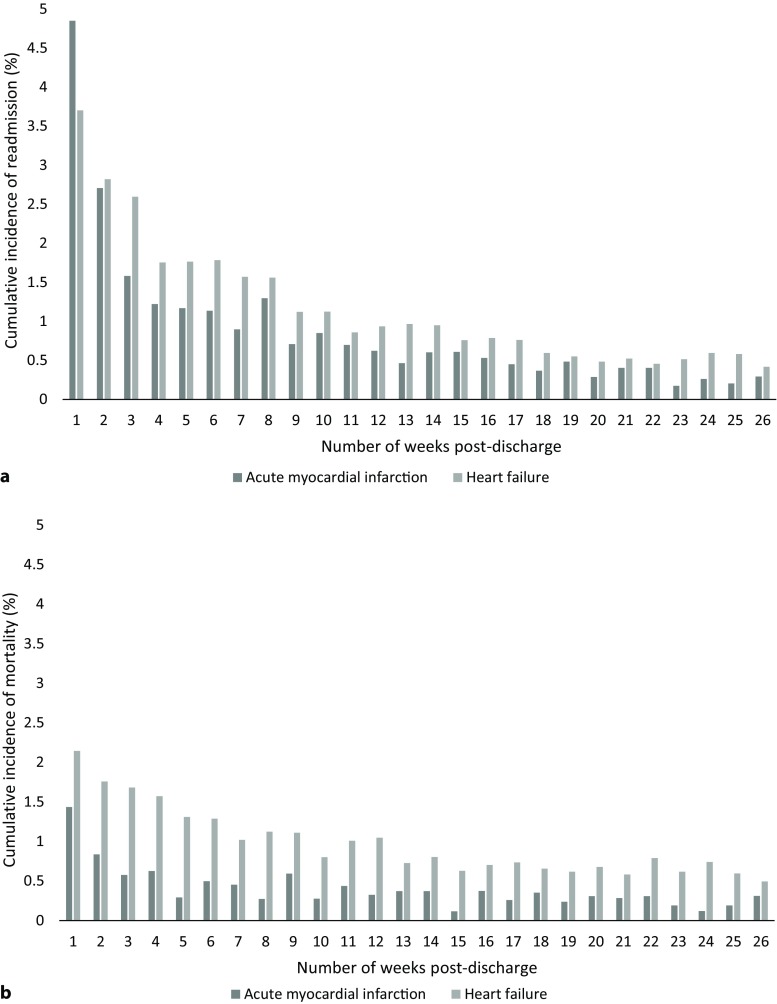
**a** The incidence rates of a first unplanned all-cause readmission within 6 months. **b** The incidence rates of mortality within 6 months (The cumulative incidence was calculated for each week post-discharge by dividing the number of readmissions and deaths by the number of patients at risk for each week until 6 months post-discharge)

Fig. 1a shows the cumulative incidences of a first unplanned all-cause readmission within 6 months post-discharge. In total, 20.4% of AMI patients (*n* = 937) and 24.6% of HF patients (*n* = 2,103) had been readmitted. The highest incidences were found in week 1: 4.8% (AMI) and 3.7% (HF) were readmitted, respectively. After week 3, the cumulative weekly incidences were lower than 2%.

### Cumulative incidence of mortality

Fig. 1b shows the cumulative incidences of mortality within 6 months post-discharge. In total, 9.9% of AMI patients (*n* = 457) and 22.4% of HF patients (*n* = 1,914) had died. The highest cumulative incidences were found in week 1: 1.4% (AMI) and 2.1% (HF) died, respectively. After week 1, the cumulative incidence of mortality in AMI patients was lower than 1%. In HF patients, a more gradual decline in cumulative incidence was found with incidences between 1.5% and 0.5% from week 4 onward.

### Risk factors of a first unplanned all-cause readmission

In AMI patients, the associations between risk factors and readmission did not vary over time. Therefore, the analyses resulted in the same model for all time windows (Table S1).

**Fig. 2:**
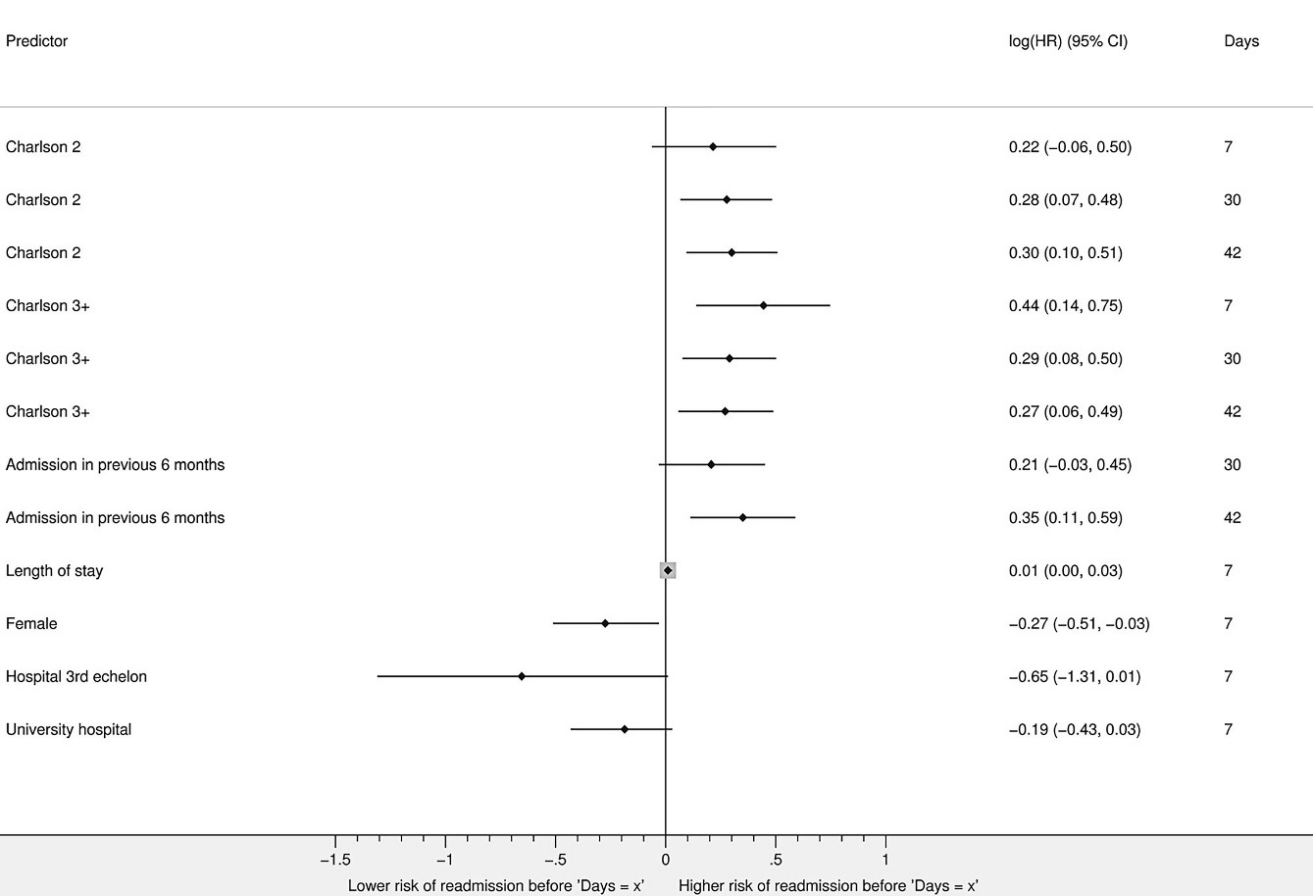
Risk factors for a first unplanned all-cause readmission whose effects change over time in heart failure patients (Hazard ratios (HRs) are displayed on a logarithmic scale to enhance compact visualisation of scattered estimates. The exact HRs are shown in Table S2, *Charlson 2* Charlson Comorbidity Index of 2, *Charlson* *3+* Charlson Comorbidity Index of ≥3)

In HF patients, a higher Charlson Comorbidity Index (CCI) increased the risk of early readmission within 7, 30 and 42 days, e. g. patients with a CCI ≥3 had a 56% greater risk of readmission within 7 days (HR 1.56, 95% confidence interval (CI) 1.15–2.11) than patients with a CCI of 1 (reference category). Women had a 24% lower risk of readmission within 7 days (HR 0.76, 95% CI 0.60–0.97) than men. Patients with an admission in the previous 6 months before the index hospitalisation had no greater risk of readmission within 30 days (HR 1.23, 95% CI 0.97–1.57) than those without such previous admissions, while a 42% greater risk was found for readmission within 42 days (HR 1.42, 95% CI 1.12–1.80) (Fig. 2, Table S2).

### Risk factors for mortality

**Fig. 3:**
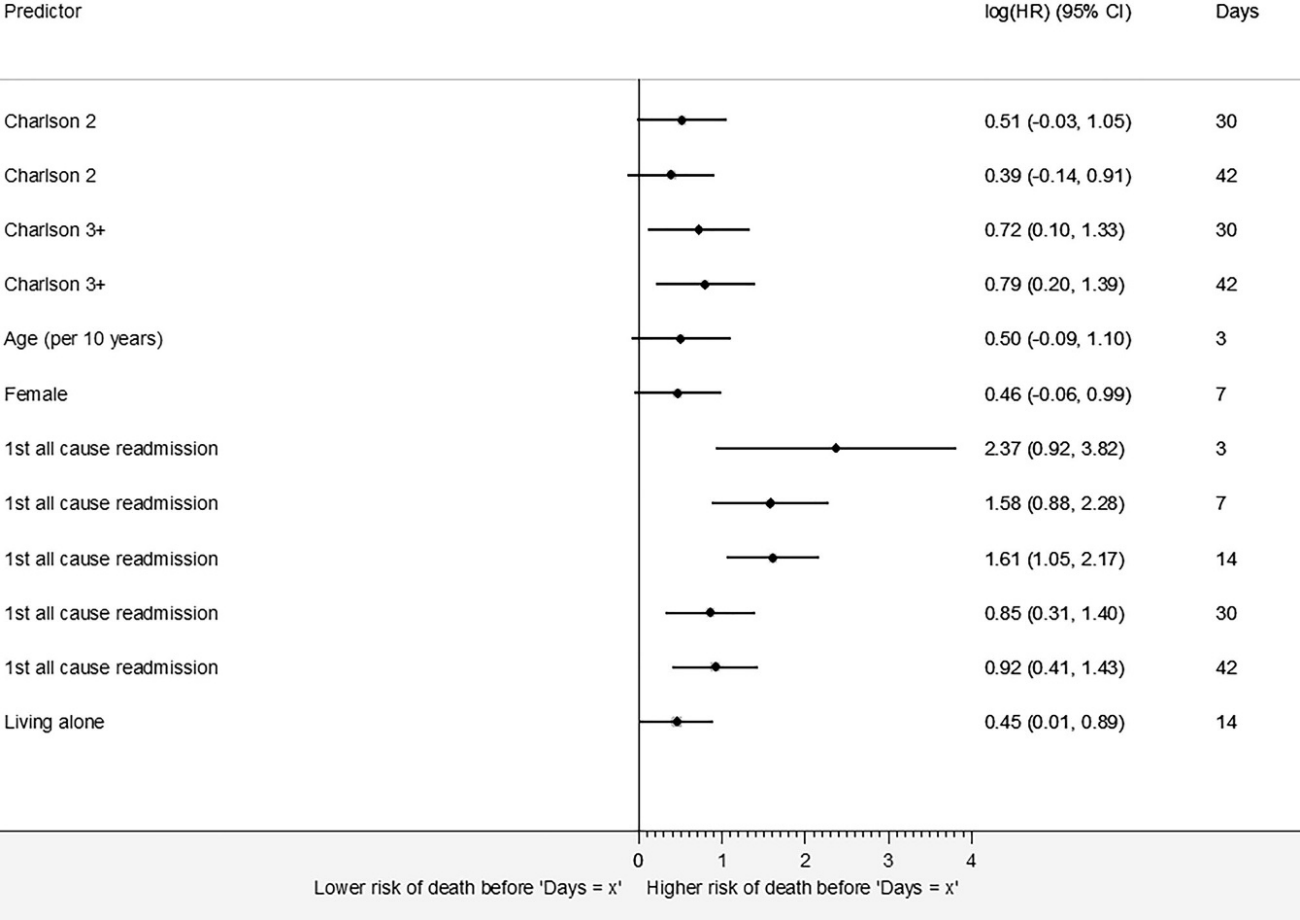
Risk factors for mortality whose effects change over time in acute myocardial infarction patients (HRs are displayed on a logarithmic scale to enhance compact visualisation of scattered estimates. The exact HRs are shown in Table S3, *Charlson* *2* Charlson Comorbidity Index of 2, *Charlson* *3+* Charlson Comorbidity Index of ≥3 *All-cause readmission.* This covariate indicates the first all-cause readmission after the index admission)

Fig. 3 (and Table S3) shows the extended Cox regression analyses of early mortality post-discharge in AMI patients. Patients living alone had a 57% greater risk of mortality within 14 days (HR 1.57, 95% CI 1.01–2.44). Patients with a CCI ≥3 had a 121% greater risk of mortality within 42 days (HR 2.21, 95% CI 1.22–4.02) than those with a CCI of 1.

**Fig. 4:**
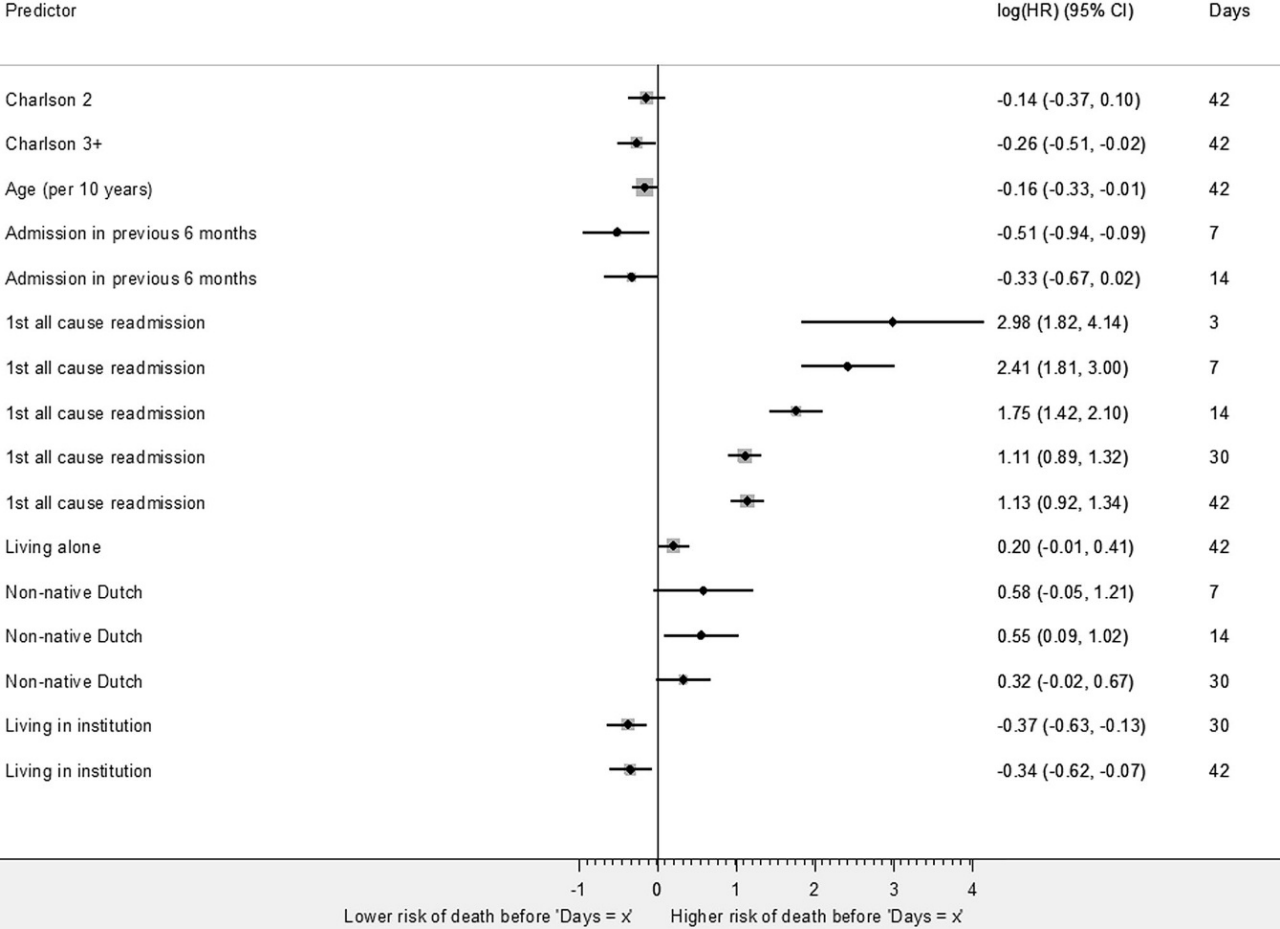
Risk factors for mortality whose effects change over time in older heart failure patients (HRs are displayed on a logarithmic scale to enhance compact visualisation of scattered estimates. The exact HRs are shown in Table S4, *Charlson* *2* Charlson Comorbidity Index of 2, *Charlson* *3+* Charlson Comorbidity Index of ≥3 *All-cause readmission.* This covariate indicates the first all-cause readmission after the index admission)

In HF patients, risk factor-time interactions were found for early mortality in all time windows (Fig. 4, Table S4). The risk factor-time interaction for readmission indicated an increased risk of mortality in all time windows. Non-native Dutch patients, compared to native Dutch, had a 74% greater risk of early mortality within 14 days (HR 1.74, 95% CI 1.09–2.78). A 15% lower risk of early mortality within 42 days was found for every 10 years of age (HR 0.85, 95% CI 0.72–0.99). Lower risks of early mortality were also found for patients living in an institution or with an admission in the previous 6 months.

## Discussion

In this retrospective cohort study of older cardiac patients after an unplanned hospital admission in the Netherlands, we found that 20.4% (AMI) and 24.6% (HF) had an unplanned all-cause readmission and 9.9% (AMI) and 22.4% (HF) had died within 6 months post-discharge. The highest incidences were found in the 1st week post-discharge. Patients with comorbidities, an admission in the previous 6 months, patients living alone and non-native Dutch patients were at highest risk of early readmission and mortality.

Consistent with the literature from the United States [13–15], this study on older Dutch cardiac patients confirms that the highest readmission and mortality rates were found right after discharge and that risks were higher and prolonged in HF patients compared to AMI patients [15]. These results suggest that the needs of older cardiac patients are insufficiently fulfilled in the early period post-discharge. The average start of TCIs after 8 days post-discharge [10] might already be too late to have a preventive effect on early readmission and mortality. Therefore, the timing of TCIs may need improvement.

We found that higher CCIs increased the risk of early readmission (HF) and mortality (AMI) at several time points. During hospital admission, older cardiac patients mainly receive disease-oriented treatments based on disease-specific guidelines, which are in turn based on studies that commonly exclude older and multimorbid patients [16, 17]. However, older cardiac patients often suffer from multiple comorbidities including diabetes, chronic pulmonary disease and renal failure [4, 5, 18]. Donzé et al. [19] found that the focus on acute illness during admission may lead to insufficient monitoring of comorbidities and increase the risk of exacerbations post-discharge. A broader assessment of older cardiac patients’ needs during hospital admission might be required [20].

### Strengths and limitations

One of the strengths of our study is that we used a large nationwide database and had the opportunity to link and combine hospital and sociodemographic data with 1‑year follow-up. This resulted in fairly rich data to examine risk factors for readmission and mortality. To our knowledge, our study is the first to examine change in those effects over time. While 11% of the cases were excluded because no linkage between hospital and sociodemographic data was possible, previous research from Statistics Netherlands showed that the number of linkable admissions were reliable for statistical analyses [21].

This study also has limitations. First, we had access only to the registries of Statistics Netherlands in 2008 and 2009 for the follow-up. Due to national trends, the incidences of readmission and mortality might nowadays have increased in HF patients and decreased in AMI patients [22]. Although the incidences might be different, we expect that the highest incidences are still found in the 1st week post-discharge. Second, the LMR contained only administrative data which precluded adjustment for cardiovascular and geriatric risk factors that are known to increase the risk of readmission and mortality (e. g. history of cardiovascular disease, disability and polypharmacy). Third, we were unable to adjust for competing risk in patients that had died before experiencing an unplanned all-cause readmission which might have resulted in an underestimation for readmission [23]. Finally, the CCIs in our data may be too low because of the underreporting of comorbidities in medical files. This may have caused an underestimation of the effect on readmission and mortality.

### Implications of findings

Hospitalised high-risk older cardiac patients need to be identified as soon as possible to guide them during care transitions. Instead of single disease-oriented treatments, a broad view on older cardiac patients’ needs is necessary [20]. Around discharge, adequate communication between hospital and community care providers, e. g. accurate and timely discharge letters, and continuity of care after discharge have proven to reduce readmissions [24]. In addition, careful assessment of patients’ readiness for discharge might be needed, as some high-risk patients might even be discharged before stable recovery [25].

While single disease-oriented interventions during hospital admission are not suitable in older cardiac patients [16, 17, 19], disease management interventions might be integrated in TCIs. More disease-specific guidance after discharge, e. g. symptom monitoring, medication reconciliation and specific guidance in medication and lifestyle adherence, might also help to reduce the risk of readmission and mortality [8, 26]. Personalised interventions might be required as HF patients were at higher and prolonged risk compared to AMI patients, and risk factors for readmission and mortality changed over time. Although readmission diagnoses are heterogeneous, early detection and proactive interventions might limit complications [13, 27].

## Conclusion

The incidences of unplanned all-cause readmission and mortality in older AMI and HF patients were highest in the 1st week post-discharge, and the effects of several risk factors for these events at discharge changed over time. Transitional care interventions need to be provided as soon as possible in admitted high-risk older patients with AMI or HF to prevent early readmission and mortality.

## Caption Electronic Supplementary Material

**Table S1** Extended Cox regression analysis of the 1st unplanned all-cause readmission in patients with acute myocardial infarction

**Table S2 **Extended Cox regression analysis of the 1st unplanned all-cause readmission in patients with heart failure (Example of interpretation of the extended Cox regression analysis: heart failure (HF) patients with a Charlson Comorbidity Index ≥3 had a 1.56 times higher risk of a first unplanned all-cause readmission within 7 days than HF patients with a Charlson Comorbidity Index of 1 (ref))

**Table S3 **Extended Cox regression analysis of mortality in patients with acute myocardial infarction (Example of interpretation of the extended Cox regression analysis: acute myocardial infarction patients living alone had a 1.57 higher risk of mortality within 14 days than patients not living alone)

**Table S4 **Extended Cox regression analysis of mortality in patients with heart failure (Example of interpretation of the extended Cox regression analysis: non-native Dutch heart failure patients had a 1.79 higher risk of mortality within 7 days than native Dutch heart failure patients)
