# Supplementary material for: Readmission and mortality in patients ≥70 years with acute myocardial infarction or heart failure in the Netherlands: a retrospective cohort study of incidences and changes in risk factors over time
Source: Neth Heart J. 2019 Feb 4;27(3):134–41. doi: 10.1007/s12471-019-1227-4 (PMC6393584; doi:10.1007/s12471-019-1227-4)
Supplement: Supplementary file 1 — Table S1 Extended Cox regression analysis of the 1st unplanned all-cause readmission in patients with acute myocardial infarction [file 12471_2019_1227_MOESM1_ESM.docx]

**S1 Table. Extended Cox regression analysis of the first unplanned all-cause readmission in patients with acute myocardial infarction**

|  | 3-days | | 7-days | | 14-days |  | 30-days | | 42-days | |
| --- | --- | --- | --- | --- | --- | --- | --- | --- | --- | --- |
|  | HR (95% CI) | p-value | HR (95% CI) | p-value | HR (95% CI) | p-value | HR (95% CI) | p-value | HR (95% CI) | p-value |
| Women | 1.07 (0.93 - 1.23) | 0.339 | 1.07 (0.93 - 1.23) | 0.339 | 1.07 (0.93 - 1.23) | 0.339 | 1.07 (0.93 - 1.23) | 0.339 | 1.07 (0.93 - 1.23) | 0.339 |
| Age per 10 years | 1.20 (1.06 - 1.33) | 0.003 | 1.20 (1.06 - 1.33) | 0.003 | 1.20 (1.06 - 1.33) | 0.003 | 1.20 (1.06 - 1.33) | 0.003 | 1.20 (1.06 - 1.33) | 0.003 |
| Non-native Dutch | 1.12 (0.92 - 1.37) | 0.260 | 1.12 (0.92 - 1.37) | 0.260 | 1.12 (0.92 - 1.37) | 0.260 | 1.12 (0.92 - 1.37) | 0.260 | 1.12 (0.92 - 1.37) | 0.260 |
| *Charlson comorbidity index* [28] |  |  |  |  |  |  |  |  |  |  |
| Score 1 (Ref) | Ref | Ref | Ref | Ref | Ref | Ref | Ref | Ref | Ref | Ref |
| Score 2 | 39.77 (30.09 - 52.56) | < 0.001 | 39.77 (30.09 - 52.56) | < 0.001 | 39.77 (30.09 - 52.56) | < 0.001 | 39.77 (30.09 - 52.56) | < 0.001 | 39.77 (30.09 - 52.56) | < 0.001 |
| Score > 3 | 44.87 (33.39 - 60.30) | < 0.001 | 44.87 (33.39 - 60.30) | < 0.001 | 44.87 (33.39 - 60.30) | < 0.001 | 44.87 (33.39 - 60.30) | < 0.001 | 44.87 (33.39 - 60.30) | < 0.001 |
| Living alone | 0.92 (0.79 - 1.07) | 0.267 | 0.92 (0.79 - 1.07) | 0.267 | 0.92 (0.79 - 1.07) | 0.267 | 0.92 (0.79 - 1.07) | 0.267 | 0.92 (0.79 - 1.07) | 0.267 |
| Living in an institution | 0.71 (0.54 - 0.94) | 0.016 | 0.71 (0.54 - 0.94) | 0.016 | 0.71 (0.54 - 0.94) | 0.016 | 0.71 (0.54 - 0.94) | 0.016 | 0.71 (0.54 - 0.94) | 0.016 |
| Annual income < €16,801 | 0.98 (0.86 - 1.12) | 0.789 | 0.98 (0.86 - 1.12) | 0.789 | 0.98 (0.86 - 1.12) | 0.789 | 0.98 (0.86 - 1.12) | 0.789 | 0.98 (0.86 - 1.12) | 0.789 |
| Length of stay | 0.99 (0.99 - 1.00) | 0.123 | 0.99 (0.99 - 1.00) | 0.123 | 0.99 (0.99 - 1.00 | 0.123 | 0.99 (0.99 - 1.00 | 0.123 | 0.99 (0.99 - 1.00 | 0.123 |
| Admission in the previous 6 months | 0.89 (0.67 - 1.18) | 0.412 | 0.89 (0.67 - 1.18) | 0.412 | 0.89 (0.67 - 1.18) | 0.412 | 0.89 (0.67 - 1.18) | 0.412 | 0.89 (0.67 - 1.18) | 0.412 |
| *Type of hospital* |  |  |  |  |  |  |  |  |  |  |
| General hospital (ref) | Ref | Ref | Ref | Ref | Ref | Ref | Ref | Ref | Ref | Ref |
| Tertiary referral hospital | 0.87 (0.76 - 0.99) | 0.033 | 0.87 (0.76 - 0.99) | 0.033 | 0.87 (0.76 - 0.99) | 0.033 | 0.87 (0.76 - 0.99) | 0.033 | 0.87 (0.76 - 0.99) | 0.033 |
| University hospital | 0.67 (0.48 - 0.92) | 0.014 | 0.67 (0.48 - 0.92) | 0.014 | 0.67 (0.48 - 0.92) | 0.014 | 0.67 (0.48 - 0.92) | 0.014 | 0.67 (0.48 - 0.92) | 0.014 |
|  |  |  |  |  |  |  |  |  |  |  |
| ***Time-depended predictors*** | None |  | None |  | None |  | None |  | None |  |
