# Supplementary material for: Readmission and mortality in patients ≥70 years with acute myocardial infarction or heart failure in the Netherlands: a retrospective cohort study of incidences and changes in risk factors over time
Source: Neth Heart J. 2019 Feb 4;27(3):134–41. doi: 10.1007/s12471-019-1227-4 (PMC6393584; doi:10.1007/s12471-019-1227-4)
Supplement: Supplementary file 2 — Table S2 Extended Cox regression analysis of the 1st unplanned all-cause readmission in patients with heart failure (Example of interpretation of the extended Cox regression analysis: heart failure (HF) patients with a Charlson Comorbidity Index ≥3 had a 1.56 times higher risk of a first unplanned all-cause readmission within 7 days than HF patients with a Charlson Comorbidity Index of 1 (ref)) [file 12471_2019_1227_MOESM2_ESM.docx]

**S2 Table. Extended Cox regression analysis of the first unplanned all-cause readmission in patients with heart failure^a^**

|  | 3-days | | 7-days | | 14-days |  | 30-days | | 42-days | |
| --- | --- | --- | --- | --- | --- | --- | --- | --- | --- | --- |
|  | HR (95% CI) | p-value | HR (95% CI) | p-value | HR (95% CI) | p-value | HR (95% CI) | p-value | HR (95% CI) | p-value |
| Women | 0.99 (0.90 - 1.09) | 0.835 | 0.95 (0.86 - 1.05) | 0.325 | 0.99 (0.90 - 1.09) | 0.835 | 0.99 (0.90 - 1.09) | 0.838 | 0.99 (0.90 - 1.09) | 0.837 |
| Age per 10 years | 1.06 (0.99 - 1.15) | 0.103 | 1.06 (0.99 - 1.15) | 0.103 | 1.06 (0.99 - 1.15) | 0.103 | 1.06 (0.99 - 1.15) | 0.102 | 1.06 (0.99 - 1.15) | 0.104 |
| Non-native Dutch | 0.93 (0.81 - 1.07) | 0.312 | 0.93 (0.81 - 1.07) | 0.309 | 0.93 (0.81 - 1.07) | 0.312 | 0.93 (0.81 - 1.07) | 0.307 | 0.93 (0.81 - 1.07) | 0.304 |
| *Charlson comorbidity index* [28] |  |  |  |  |  |  |  |  |  |  |
| Score 1 (Ref) | Ref | Ref | Ref | Ref | Ref | Ref | Ref | Ref | Ref | Ref |
| Score 2 | 2.69 (2.43 - 2.98) | < 0.001 | 2.78 (2.49 - 3.11) | < 0.001 | 2.69 (2.43 - 2.98) | < 0.001 | 3.03 (2.64 - 3.46) | < 0.001 | 3.15 (2.72 - 3.66) | < 0.001 |
| Score > 3 | 3.91 (3.51 - 4.36) | < 0.001 | 4.18 (3.72 - 4.70) | < 0.001 | 3.91 (3.51 - 4.36) | < 0.001 | 4.44 (3.84 - 5.12) | < 0.001 | 4.53 (3.86 - 5.31) | < 0.001 |
| Living alone | 0.95 (0.86 - 1.06) | 0.369 | 0.96 (0.86 - 1.06) | 0.377 | 0.95 (0.86 - 1.06) | 0.369 | 0.95 (0.86 - 1.06) | 0.370 | 0.96 (0.86 - 1.06) | 0.373 |
| Living in an institution | 1.01 (0.88 - 1.16) | 0.882 | 1.01 (0.88 - 1.16) | 0.893 | 1.01 (0.88 - 1.16) | 0.882 | 1.01 (0.88 - 1.16) | 0.886 | 1.01 (0.88 - 1.16) | 0.883 |
| Annual income < €16,801 | 0.87 (0.80 - 0.96) | 0.003 | 0.88 (0.80 - 0.96) | 0.003 | 0.87 (0.80 - 0.96) | 0.003 | 0.87 (0.80 - 0.96) | 0.003 | 0.87 (0.80 - 0.96) | 0.003 |
| Length of stay | 1.01 (0.997 - 1.006) | 0.631 | 1.00 (0.998 - 1.01) | 0.233 | 1.01 (0.997 - 1.006) | 0.631 | 1.00 (0.997 - 1.006) | 0.640 | 1.00 (0.997 - 1.006) | 0.645 |
| Admission in the previous 6 months | 1.62 (1.44 - 1.82) | < 0.001 | 1.62 (1.44 - 1.83) | < 0.001 | 1.62 (1.44 - 1.82) | < 0.001 | 1.78 (1.52 - 2.07) | < 0.001 | 1.95 (1.65 - 2.30) | < 0.001 |
| *Type of hospital* |  |  |  |  |  |  |  |  |  |  |
| General hospital (ref) | Ref | Ref | Ref | Ref | Ref | Ref | Ref | Ref | Ref | Ref |
| Tertiary referral hospital | 0.95 (0.87 - 1.04) | 0.271 | 0.93 (0.84 - 1.02) | 0.113 | 0.95 (0.87 - 1.04) | 0.271 | 0.95 (0.87 - 1.04) | 0.269 | 0.95 (0.87 - 1.04) | 0.266 |
| University hospital | 0.60 (0.46 - 0.79) | < 0.001 | 0.54 (0.40 - 0.73) | < 0.001 | 0.60 (0.46 - 0.79) | < 0.001 | 0.60 (0.46 - 0.78) | < 0.001 | 0.60 (0.46 - 0.78) | < 0.001 |
|  |  |  |  |  |  |  |  |  |  |  |
| ***Time-depended predictors*** | None |  |  |  | None |  |  |  |  |  |
| Women |  |  | 0.76 (0.60 - 0.97) | 0.028 |  |  |  |  |  |  |
| Age per 10 years |  |  |  |  |  |  |  |  |  |  |
| Non-native Dutch |  |  |  |  |  |  |  |  |  |  |
| *Charlson comorbidity index* [28] |  |  |  |  |  |  |  |  |  |  |
| Score 1 (Ref) |  |  | Ref | Ref |  |  | Ref | Ref | Ref | Ref |
| Score 2 |  |  | 1.24 (0.94 - 1.65) | 0.128 |  |  | 1.32 (1.07 - 1.62) | 0.009 | 1.35 (1.10 - 1.66) | 0.004 |
| Score > 3 |  |  | 1.56 (1.15 - 2.11) | 0.004 |  |  | 1.33 (1.08 - 1.65) | 0.008 | 1.31 (1.06 - 1.63) | 0.012 |
| Living alone |  |  |  |  |  |  |  |  |  |  |
| Living in an institution |  |  |  |  |  |  |  |  |  |  |
| Annual income < €16,801 |  |  |  |  |  |  |  |  |  |  |
| Length of stay |  |  |  |  |  |  |  |  |  |  |
| Admission in the previous 6 months |  |  |  |  |  |  | 1.23 (0.97 - 1.57) | 0.086 | 1.42 (1.12 - 1.80) | 0.004 |
| *Type of hospital* |  |  |  |  |  |  |  |  |  |  |
| General hospital (ref) |  |  | Ref | Ref |  |  |  |  |  |  |
| Tertiary referral hospital |  |  | 0.52 (0.27 - 1.01) | 0.128 |  |  |  |  |  |  |
| University hospital |  |  | 0.83 (0.65 - 1.03) | 0.053 |  |  |  |  |  |  |

^a^ Example of interpretation of the Extended Cox regression analysis: Heart failure patients with a Charlson comorbidity index > 3 had a 1.56 times higher hazard of a first unplanned all-cause readmission within 7 days than heart failure patients with a Charlson comorbidity index of 1 (ref)
