# Supplementary material for: Readmission and mortality in patients ≥70 years with acute myocardial infarction or heart failure in the Netherlands: a retrospective cohort study of incidences and changes in risk factors over time
Source: Neth Heart J. 2019 Feb 4;27(3):134–41. doi: 10.1007/s12471-019-1227-4 (PMC6393584; doi:10.1007/s12471-019-1227-4)
Supplement: Supplementary file 3 — Table S3 Extended Cox regression analysis of mortality in patients with acute myocardial infarction (Example of interpretation of the extended Cox regression analysis: acute myocardial infarction patients living alone had a 1.57 higher risk of mortality within 14 days than patients not living alone) [file 12471_2019_1227_MOESM3_ESM.docx]

**S3 Table. Extended Cox regression analysis of mortality in patients with acute myocardial infarction^a^**

|  | 3-days | | 7-days | | 14-days |  | 30-days | | 42-days | |
| --- | --- | --- | --- | --- | --- | --- | --- | --- | --- | --- |
|  | HR (95% CI) | p-value | HR (95% CI) | p-value | HR (95% CI) | p-value | HR (95% CI) | p-value | HR (95% CI) | p-value |
| Women | 0.91 (0.75 - 1.12) | 0.369 | 0.97 (0.79 - 1.21) | 0.807 | 0.91 (0.75 - 1.12) | 0.369 | 0.91 (0.75 - 1.14) | 0.366 | 0.91 (0.75 - 1.12) | 0.368 |
| Age per 10 years | 2.52 (2.12 - 2.97) | < 0.001 | 2.43 (2.06 - 2.87) | < 0.001 | 2.43 (2.07 - 2.87) | < 0.001 | 2.43 (2.08 - 2.87) | < 0.001 | 2.43 (2.08 - 2.87) | < 0.001 |
| Non-native Dutch | 1.27 (0.96 - 1.67) | 0.094 | 1.27 (0.96 - 1.67) | 0.093 | 1.26 (0.96 - 1.67) | 0.097 | 1.27 (0.96 - 1.67) | 0.094 | 1.27 (0.96 - 1.67) | 0.095 |
| *Charlson comorbidity index* [28[ |  |  |  |  |  |  |  |  |  |  |
| Score 1 (Ref) | Ref | Ref | Ref | Ref | Ref | Ref | Ref | Ref | Ref | Ref |
| Score 2 | 1.26 (0.97 - 1.64) | 0.082 | 1.26 (0.97 - 1.63) | 0.085 | 1.26 (0.97 - 1.64) | 0.082 | 1.56 (1.12 - 2.18) | 0.009 | 1.55 (1.08 - 2.22) | 0.017 |
| Score > 3 | 2.19 (1.65 - 2.92) | < 0.001 | 2.19 (1.65 - 2.92) | < 0.001 | 2.20 (1.65 - 2.93) | < 0.001 | 2.87 (2.01 - 4.10) | < 0.001 | 3.08 (2.11 - 4.49) | < 0.001 |
| Living alone | 1.00 (0.81 - 1.25) | 0.976 | 1.00 (0.81 - 1.25) | 0.981 | 1.11 (0.87 - 1.41) | 0.396 | 1.00 (0.81 - 1.25) | 0.984 | 1.00 (0.81 - 1.25) | 0.986 |
| Living in an institution | 0.84 (0.60 - 1.17) | 0.299 | 0.84 (0.60 - 1.17) | 0.303 | 0.84 (0.60 - 1.17) | 0.306 | 0.84 (0.60 - 1.17) | 0.299 | 0.84 (0.60 - 1.17) | 0.301 |
| Annual income < €16,801 | 0.78 (0.64 - 0.94) | 0.008 | 0.78 (0.64 - 0.94) | 0.008 | 0.78 (0.64 - 0.94) | 0.008 | 0.77 (0.64 - 0.94) | 0.008 | 0.77 (0.64 - 0.94) | 0.008 |
| Length of stay | 1.02 (1.01 - 1.03) | < 0.001 | 1.02 (1.01 - 1.03) | < 0.001 | 1.02 (1.01 - 1.03) | < 0.001 | 1.02 (1.01 - 1.03) | < 0.001 | 1.02 (1.01 - 1.03) | < 0.001 |
| Admission in the previous 6 months | 1.21 (0.82 - 1.79) | 0.345 | 1.21 (0.82 - 1.79) | 0.344 | 1.21 (0.82 - 1.79) | 0.344 | 1.21 (0.81 - 1.79) | 0.351 | 1.20 (0.81 - 1.79) | 0.356 |
| *Type of hospital* |  |  |  |  |  |  |  |  |  |  |
| General hospital (ref) | Ref | Ref | Ref | Ref |  |  |  |  |  |  |
| Tertiary referral hospital | 0.84 (0.70 - 1.02) | 0.077 | 0.84 (0.70 - 1.02) | 0.077 | 0.84 (0.70 - 1.02) | 0.074 | 0.84 (0.70 - 1.02) | 0.074 | 0.84 (0.69 - 1.02) | 0.073 |
| University hospital | 0.84 (0.53 - 1.31) | 0.438 | 0.84 (0.53 - 1.32) | 0.442 | 0.84 (0.53 - 1.32) | 0.444 | 0.84 (0.53 - 1.32) | 0.434 | 0.84 (0.53 - 1.32) | 0.436 |
| First all-cause readmission with 6 months | 2.04 (1.59 - 2.61) | < 0.001 | 2.22 (1.72 - 2.86) | < 0.001 | 2.50 (1.93 - 3.25) | < 0.001 | 2.34 (1.77 - 3.19) | < 0.001 | 2.58 (1.89- 3.54) | < 0.001 |
| ***Time-depended predictors*** |  |  |  |  |  |  |  |  |  |  |
| Women | - |  | 1.59 (0.94 - 2.70) | 0.087 | - |  | - |  | - |  |
| Age per 10 years | 1.65 (0.91 - 3.00) | 0.101 | - |  | - |  | - |  | - |  |
| Non-native Dutch | - |  | - |  | - |  | - |  | - |  |
| *Charlson comorbidity index* [28] | - |  | - |  | - |  | - |  | - |  |
| Score 1 (Ref) | - |  | - |  | - |  | - |  | - |  |
| Score 2 | - |  | - |  | - |  | 1.67 (0.97 - 2.87) | 0.066 | 1.47 (0.87 - 2.49) | 0.151 |
| Score > 3 | - |  | - |  | - |  | 2.05 (1.11 - 3.79) | 0.022 | 2.21 (1.22 - 4.02) | 0.009 |
| Living alone | - |  | - |  | 1.57 (1.01 - 2.44) | 0.044 | - |  | - |  |
| Living in an institution | - |  | - |  | - |  | - |  | - |  |
| Annual income < €16,801 | - |  | - |  | - |  | - |  | - |  |
| Length of stay | - |  | - |  | - |  | - |  | - |  |
| Admission in the previous six months | - |  | - |  | - |  | - |  | - |  |
| *Type of hospital* | - |  | - |  | - |  | - |  | - |  |
| General hospital (ref) | - |  | - |  | - |  | - |  | - |  |
| Tertiary referral hospital | - |  | - |  | - |  | - |  | - |  |
| University hospital | - |  | - |  | - |  | - |  | - |  |
| First all-cause readmission within 6 months | 10.75 (2.51 - 45.45) | 0.001 | 4.85 (2.40 - 9.80) | < 0.001 | 5.00 (2.86 - 8.77) | < 0.001 | 2.35 (1.37 - 4.05) | 0.002 | 2.50 (1.50 - 4.17) | < 0.001 |

^a^ Example of interpretation of the Extended Cox regression analysis: Acute myocardial infarction patients living alone had a 1.57 higher hazard of mortality within 14 days than patients not living alone.
