# Supplementary material for: Readmission and mortality in patients ≥70 years with acute myocardial infarction or heart failure in the Netherlands: a retrospective cohort study of incidences and changes in risk factors over time
Source: Neth Heart J. 2019 Feb 4;27(3):134–41. doi: 10.1007/s12471-019-1227-4 (PMC6393584; doi:10.1007/s12471-019-1227-4)
Supplement: Supplementary file 4 — Table S4 Extended Cox regression analysis of mortality in patients with heart failure (Example of interpretation of the extended Cox regression analysis: non-native Dutch heart failure patients had a 1.79 higher risk of mortality within 7 days than native Dutch heart failure patients) [file 12471_2019_1227_MOESM4_ESM.docx]

**S4 Table. Extended Cox regression analysis of mortality in patients with heart failure^a^**

|  | 3-days | | 7-days | | 14-days |  | 30-days | | 42-days | |  |
| --- | --- | --- | --- | --- | --- | --- | --- | --- | --- | --- | --- |
|  | HR (95% CI) | p-value | HR (95% CI) | p-value | HR (95% CI) | p-value | HR (95% CI) | p-value | HR (95% CI) | p-value |  |
| Women | 0.78 (0.71 - 0.87) | < 0.001 | 0.78 (0.71 - 0.87) | < 0.001 | 0.78 (0.71 - 0.87) | < 0.001 | 0.79 (0.71 - 0.87) | < 0.001 | 0.78 (0.71 - 0.87) | < 0.001 |  |
| Age per 10 years | 1.97 (1.82 - 2.11) | < 0.001 | 1.97 (1.82 - 2.11) | < 0.001 | 1.97 (1.82 - 2.11) | < 0.001 | 1.97 (1.82 - 2.11) | < 0.001 | 1.84 (1.66 - 2.04) | < 0.001 |  |
| Non-native Dutch | 0.94 (0.81 - 1.09) | 0.424 | 0.98 (0.84 - 1.15) | 0.835 | 1.02 (0.86 - 1.20) | 0.841 | 1.03 (0.86 - 1.23) | 0.725 | 0.94 (0.80 - 1.09) | 0.403 |  |
| *Charlson comorbidity index* [28] |  |  |  |  |  |  |  |  |  |  |  |
| Score 1 (Ref) | Ref | Ref | Ref | Ref | Ref | Ref | Ref | Ref | Ref | Ref |  |
| Score 2 | 1.04 (0.92 - 1.16) | 0.545 | 1.04 (0.92 - 1.16) | 0.555 | 1.04 (0.92 - 1.16) | 0.563 | 1.04 (0.92 - 1.16) | 0.574 | 0.97 (0.84 - 1.13) | 0.717 |  |
| Score > 3 | 1.47 (1.30 - 1.66) | < 0.001 | 1.47 (1.30 - 1.66) | < 0.001 | 1.47 (1.30 - 1.66) | < 0.001 | 1.47 (1.30 - 1.66) | < 0.001 | 1.32 (1.12 - 1.55) | 0.001 |  |
| Living alone | 0.88 (0.79 - 0.99) | 0.027 | 0.89 (0.79 - 0.99) | 0.029 | 0.89 (0.80 - 0.99) | 0.030 | 0.89 (0.80 - 0.99) | 0.032 | 0.97 (0.84 - 1.11) | 0.612 |  |
| Living in an institution | 0.94 (0.82 - 1.08) | 0.380 | 0.94 (0.82 - 1.08) | 0.374 | 0.94 (0.82 - 1.08) | 0.361 | 0.82 (0.69 - 0.97) | 0.018 | 0.81 (0.67 - 0.97) | 0.019 |  |
| Annual income < €16,801 | 0.72 (0.66- 0.80) | < 0.001 | 0.73 (0.66- 0.80) | < 0.001 | 0.72 (0.66- 0.80) | < 0.001 | 0.72 (0.66- 0.80) | < 0.001 | 0.72 (0.66 - 0.80) | < 0.001 |  |
| Length of stay | 1.02 (1.02 - 1.03) | < 0.001 | 1.02 (1.02 - 1.03) | < 0.001 | 1.02 (1.02 - 1.03) | < 0.001 | 1.02 (1.02 - 1.03) | < 0.001 | 1.02 (1.02 - 1.03) | < 0.001 |  |
| Admission in the previous 6 months | 1.22 (1.07 - 1.40) | 0.004 | 1.16 (1.01 - 1.34) | 0.043 | 1.15 (0.99 - 1.34) | 0.063 | 1.22 (1.06 - 1.39) | 0.004 | 1.22 (1.06 - 1.40) | 0.004 |  |
| *Type of hospital* |  |  |  |  |  |  |  |  |  |  |  |
| General hospital (ref) |  |  |  |  |  |  |  |  |  |  |  |
| Tertiary referral hospital | 0.99 (0.90 - 1.08) | 0.742 | 0.98 (0.90 - 1.08) | 0.732 | 0.98 (0.90 - 1.08) | 0.730 | 0.99 (0.90 - 1.08) | 0.740 | 0.99 (0.90 - 1.08) | 0.766 |  |
| University hospital | 1.01 (0.78 - 1.30) | 0.968 | 1.01 (0.78 - 1.30) | 0.973 | 1.01 (0.78 - 1.30) | 0.971 | 1.01 (0.78 - 1.30) | 0.958 | 1.01 (0.78 - 1.30) | 0.966 |  |
| Readmission | 1.90 (1.72 - 2.11) | < 0.001 | 2.05 (1.86 - 2.28) | < 0.001 | 2.24 (2.02 - 2.49) | < 0.001 | 2.47 (2.20 - 2.77) | < 0.001 | 2.77 (2.44 - 3.14) | < 0.001 |  |
| ***Time-depended predictors*** |  |  |  |  |  |  |  |  |  |  |  |
| Women | - |  |  |  | - |  | - |  |  |  |  |
| Age per 10 years | - |  | - |  | - |  | - |  | 0.85 (0.72 - 0.99) | 0.042 |  |
| Non-native Dutch | - |  | 1.79 (0.95 - 3.36) | 0.070 | 1.74 (1.09 - 2.78) | 0.022 | 1.38 (0.98 - 1.95) | 0.063 |  |  |  |
| *Charlson comorbidity index* [28] | - |  | - |  | - |  | - |  | - |  |  |
| Score 1 (Ref) | - |  | - |  | - |  | - |  | - |  |  |
| Score 2 | - |  | - |  | - |  | - |  | 0.87 (0.69 - 1.10) | 0.237 |  |
| Score > 3 | - |  | - |  | - |  | - |  | 0.77 (0.60 - 0.98) | 0.031 |  |
| Living alone | - |  | - |  | - |  | - |  | 1.22 (0.99 - 1.50) | 0.060 |  |
| Living in an institution | - |  | - |  | - |  | 0.69 (0.53 - 0.88) | 0.003 | 0.71 (0.54 - 0.93) | 0.012 |  |
| Annual income < €16,801 | - |  | - |  | - |  | - |  | - |  |  |
| Length of stay | - |  | - |  |  |  | - |  | - |  |  |
| Admission in the previous 6 months | - |  | 0.60 (0.39 - 0.91) | 0.018 | 0.72 (0.51 - 1.02) | 0.064 | - |  | - |  |  |
| *Type of hospital* | - |  | - |  | - |  | - |  | - |  |  |
| General hospital (ref) | - |  | - |  | - |  | - |  | - |  |  |
| Tertiary referral hospital | - |  | - |  | - |  | - |  | - |  |  |
| University hospital | - |  | - |  | - |  | - |  | - |  |  |
| First all-cause readmission within 6 months | 19.61 (6.17 - 62.50) | < 0.001 | 11.11 (6.14 - 20.00) | < 0.001 | 5.78 (4.12 - 8.13) | < 0.001 | 3.03 (2.44 - 3.76) | < 0.001 | 3.11 (2.52 - 3.83) | < 0.001 |  |

^a^ Example of interpretation of the Extended Cox regression analysis: Non-native Dutch heart failure patients had a 1.79 higher hazard of mortality within 7 days than native Dutch heart failure patients.
